# N-3 fatty acid intake altered fat content and fatty acid distribution in chicken breast muscle, but did not influence mRNA expression of lipid-related enzymes

**DOI:** 10.1186/1476-511X-13-92

**Published:** 2014-06-03

**Authors:** Anna Haug, Nicole F Nyquist, Magny Thomassen, Arne T Høstmark, Tone-Kari Knutsdatter Østbye

**Affiliations:** 1 Department of Animal and Aquacultural Sciences, The Norwegian University of Life Sciences, P.O. BOX 5003, Ås 1432, Norway; 2 Institute of Health and Society, University of Oslo, Blindern, P.O. BOX 113, Oslo 0318, Norway; 3 Nofima, Osloveien 1, P.O. BOX 210, Ås 1430, Norway

## Abstract

**Background:**

The conversions of the n-3 and n-6 fatty acid of plant origin to the C20 and C22 very long chain fatty acids (LCPUFAs) is regulated by several cellular enzymes such as elongases and desaturases.

**Methods:**

Sixty-five male one-day old chickens (Ross 308) were randomly divided into four groups and given one of four diets; with or without linseed oil (LO), (the diets contained equal amounts of fat) and with low or high selenium (Se). Final body weight, amount of Se and fat in breast muscle, fatty acid profile, and gene expression for fatty acid desaturases (Fads1, Fads2, Fads9), HMG-CoA reductase, Acyl-CoA oxidase (Acox), carnitine palmitoyl transferase1 (Cpt1), superoxide dismutase (Sod) and glutathione peroxidase4 (Gpx4) were analyzed in all animals, and Gpx activity in whole blood was determined.

**Results:**

mRNA expression of elongases and desaturases in chicken breast muscle was not affected by feed rich in C18:3n-3. The highly positive correlation between amount of fat in breast muscle and the product/precursor indices of monounsaturated fatty acid synthesis, and the negative correlation between muscle fat and indices of LCPUFA synthesis should be further studied.

**Conclusion:**

mRNA expression in chicken breast muscle of elongases and desaturases was not affected by feed rich in C18:3n-3. The highly positive correlation between amount of fat in breast muscle and the product/precursor indices of monounsaturated fatty acid synthesis, and the negative correlation between muscle fat and indices of LCPUFA synthesis should be further studied.

## Background

The conversion of the n-3 and n-6 fatty acid of plant origin alpha-linolenic acid (ALA, 18:3n-3) and linoleic acid (LA, 18:2n-6) to the C20 and C22 very long chain fatty acids (n3 LCPUFAs) eicosapentaenoic acid (EPA, 20:5n-3), docosapentaenoic acid (DPA, 22:5n-3) and docosahexaenoic acid (DHA, 22:6n-3) and the n-6 fatty acid dihomo-gamma-linolenic acid (DGLA, 20:3n-6) and arachidonic acid (AA, 20:4n-6) may have health implications since they have been linked to several life-style diseases such as cardio-metabolic and inflammatory disorders [1-3]. The elongation and desaturation of ALA to EPA, DPA and DHA, and LA to DGLA and AA in the cells involves the enzymes Delta-6 desaturase, Delta-5 desaturase and elongases. Indices of elongation and desaturation can be calculated by dividing the product fatty acid by the precursor; e.g. 18:3n-6/18:2n-6 as an estimate of Delta-6 desaturation, and these indices are giving an estimate of the ability of enzymes to elongate or desaturate the C18 fatty acids to C20 and C22 LCPUFAs. Delta-9 desaturation of saturated fatty acids to monounsaturated fatty acids (MUFAs) can be estimated by the ratios 16:1c9/16:0 and 18:1c9/18:0. However, all the indices are indirect measures, and cannot be assumed to directly reflect desaturase activities [4].

Delta-5 and Delta-6 fatty acid desaturases, encoded by fatty acid desaturase (Fads) Fads1 and Fads2, are involved in biosynthesis of PUFAs [5,6]. Delta-6 desaturase inserts double bonds in the fatty acids 18:3n-3, 24:5n-3, 18:2n-6 and 24:4n-6, and Delta-5 in 20:4n-3 and 20:3n-6. Expression and activity of these enzymes are in mammals shown to be regulated by different factors like diets and hormones. Limited information exists on chicken [7]. Delta-9 desaturase, also called Stearoyl-CoA desaturase 1 (Scd1), is involved in the synthesis of monounsaturated acyl-CoA by insertion of double bond in the Delta-9 position on substrates like palmitoleyl- and stearoyl-CoA. This enzyme is indicated as one of the major genes regulating lipid secretion [8]. HMG-CoA reductase (or 3-hydroxy-3-methyl-glutaryl-CoA reductase) is the rate-controlling enzyme in the cholesterol synthesis pathway. Because of its key role in metabolism of cholesterol, the HMG-CoA reductase is important in meat quality and animal growth performance. Acyl-Coenzyme A Oxidase 1 (Acox1) is the first enzyme of the peroxisomal fatty acid beta-oxidation pathway, which catalyzes the desaturation of acyl-CoAs to 2-trans-enoyl-CoA [9]. The mitochondrial oxidation of fatty acids is initiated by carnitine palmitoyl transferease 1 (Cpt1), which catalyzes the transfer of the acyl group of long-chain fatty acid-CoA conjugates onto carnitine, an essential step for the mitochondrial uptake of long-chain fatty acids and their subsequent beta-oxidation in the mitochondrion. In chicken, as in mammals, two different Cpt1 isoform exists, one liver and one muscle Cpt1 isoform [10].

Linseed oil (LO) is rich in alpha-linolenic acid (ALA), containing about 50% ALA [11]. LO also contains less LA than the most commonly used plant oils. LO supplementation to human diets and to feed has been shown to increase ALA and n-3 LCPUFAs, and reduce LA and AA in blood and in meat [12,13].

Se supplementation to the feed has also been shown to increase the concentration of n-3 LCPUFAs in muscle [14-16]. Additional Se intake may benefit people with low Se status [17], and Se supplementation may thus be a method to produce healthier chicken meat for the consumer.

Whether the increase in n-3 LCPUFAs in chicken breast muscle following LO supplementation to the feed is related to increased expression of genes involved in the PUFA metabolism is not known. In rats the expression of genes involved in desaturation and elongation of ALA to n-3 LCPUFA was shown not to be affected by ALA in the diet [18].

Desaturase activities have been linked to chronic diseases and health, e.g. to obesity and insulin resistance [19-21]. The causes for these diseases are complex and multifactorial, but the amount of fat in muscle cells can be underlying the state of inflammation, insulin resistance and metabolic syndrome [22].

The aim of the present study was to investigate the effects of dietary LO and Se on mRNA expression of desaturases and other enzymes involved in lipid metabolism and the antioxidant defense system, and to compare gene expressions with indices of desaturases and elongases and fat percent in chicken breast muscle.

## Methods

### Diets

Wheat based diets were given to the chickens from day 1 until termination of the animals at day 29. The chicken diets contained identical amounts of ingredients and macronutrients with exception of the supplemented Se. The Se content was either 0.13 mg/kg or 1.10 mg/kg in the low Se and high Se diets. The total fat supplement was 80 g/kg in the diets, and the content of LO was either 0 or 24 g/kg feed in the diets without or with LO (Table 1).

**Table 1:** Chicken feed composition; ingredients are listed as g/kg feed, and selenium analyzed in feed is given as mg/kg

**Linseed oil (LO)**	**Without**	**Without**	**With**	**With**
**Selenium (Se)**	**Low**	**High**	**Low**	**High**
	**n = 16**	**n = 16**	**n = 17**	**n = 16**
Fat*:				
Rendered fat	40	40	56	56
Linseed oil	0	0	24	24
Soybean oil	40	40	0	0
Wheat	450	450	450	450
Corn gluten	100	100	100	100
Soybean flour	170	170	170	170
Oat	150	150	150	150
Minor constituents**	50	50	50	50
Selenium, mg/kg	0.13	1.05	0.13	1.05

The energy content of the feed was about 19 MJ/ kg, and the E% of ALA in the feed without LO was 0.7 E%, and it was 2.5 E% ALA in the feed with LO. It was 6 E% LA in the feed without LO and 4 E% LA in the feed with LO.

*Fatty acid composition of the feed without LO: 35.6% 18:2n-6, 4.0% 18:3n-3 and n-6/n-3 = 8.7, and fatty acid composition of the feed with LO: 19.8% 18:2n-6, 14.3% 18:3n-3 and n-6/n-3 = 1.4. The percentage of 16:0, 18:0 and 18:1 were similar in the feed without LO and with LO, being about 18%, 9% and 27%, respectively.

**Histidine 0.1%, choline choride 0.13%, mono-calcium phosphate 1.4%, ground limestone 1.3%, sodium chloride 0.25%, sodium bicarbonate 0.2%, vitamin A, E, D,K,B 0.18%, L-lysine 0.4%, DL-methionine 0.2%, L-threonine 0.2%.

### Animals

The animals (65 animals) in this experiment were Ross 308 broiler chickens from Samvirkekylling (Norway). The chickens were treated in accordance with National and International guidelines concerning the use of animals in research (Norwegian Animal and Welfare Act, European Convention for the protection of Vertebrate Animals used for Experimental and other Scientific Purposes, CETS No.: 123 1986).

The animals had free access to feed and water. They were kept group wise in mesh floored battery cages from day 1 to day 12, and then they were placed in separate metabolism cages from day 12 until day 29. The birds were inspected twice daily by qualified handlers, and every other day by a veterinarian throughout the trial period.

At day 29, the animals were stunned by a sharp blow to the head and killed by exsanguinations and blood samples were collected from the carotid aorta for Gpx determination. Samples from the right breast muscle from the chickens were collected in RNA-later (Sigma-Aldrich) for determination of gene expression. The rest of the breast muscle was removed and frozen at -20°C for fatty acid analyses by gas chromatography.

### Analyses

Fatty acid composition of breast muscle and feed was determined by gas chromatography in accordance with O’Fallon et al. [23]. Percent of total fatty acids (% FAME) are presented, (mg fatty acid/g tissue wet weight, were determined but are not presented).

### Selenium

The total Se concentration in chicken breast muscle and feed was performed according to NMKL method 161, on ICP-AES instrument (Perkin Elmer, Optima 7300).

### Gpx in whole blood

Heparinized whole blood samples were stored at -20°C and analyzed for Gpx according to the method by Paglia and Valentine [24]. The analyses were performed at a Maxmat SA automatic spectrophotometer (Montpellier, France).

### Gene expression analyses (mRNA)

RNA was isolated using PureLink™ Pro 96 RNA Purification Kit with On-column DNase Digestion (Invitrogen, Carlsbad, USA). Concentration and purity of RNA were evaluated using NanoDrop 1000 Spectrophotometer (NanoDrop Technologies, USA). cDNA was made from 500 ng RNA in a 20 μL reaction volume by using AffinityScript QPCR cDNA Synthesis Kit (Agilent Technologies, CA, USA). The cDNA synthesis was run in a PCR machine under the following conditions: 25°C for 5 minutes, 42°C for 45 minutes, 95°C for 5 minutes. The reaction mix for quantitative PCR consisted of 4 μl diluted (1:10) cDNA, 1 μl forward and reverse primer (final concentration of 0.5 μM, Table 2), and 5 μl SYBR Green-I Master (Roche Applied Science, Germany). A standard curve was included for each primer pair to evaluate the primer efficiency. All samples were analyzed in parallels, and a non-template control with water substituted for cDNA was run for each primer pair. The qPCR reaction was run on a LightCycler®480 (Roche Diagnostics Gmbh, Germany) under the following conditions: 95°C for 5 minutes, 45 cycles at 95°C for 15 seconds and 60°C for 1 minutes. A melting curve analysis was run to confirm a single PCR product (95°C for 5 seconds and 65°C for 1 minute). Rpol2, gapdh, actb and ywhaz were evaluated as reference genes using RefFinder (http://www.leonxie.com/referencegene.php?type=reference). The relative gene expression level was calculated according to the ΔΔCt method [25] using rpol as reference gene.

**Table 2:** Gene expression analyses

**Gene**	**Accession no.**	**Forward (5′-3′)**	**Reverse (5′-3′)**	**Amplicon (bp)**
fads1	XM_420557	GAGCCATCGGTGAGGGTTTC	CTCCAGTCCTTTCCTTGCGT	186
fads2	NM_001160428	ACTGGTGGAACCATCGTCAC	GCAGAGGTGGGAAGATGAGG	189
fads9	NM_204890	GGAGCCCTAGGAGAAGGTTTC	AAATTGAAGCGCCAGCCAAA	86
hmgcr	NM_204485	TGTTGTAAGGCTGCCCTCTG	TAGGCGGGCAAACCTACTTG	118
cpt1	DQ314726	TGACGTCGATTTCTGCTGCT	GCAGCGCGATCTGAATGAAG	98
acox1	NM_001006205	CCAGTCAGCTTGGTAGAGGC	AGTGACAGTGTGCCTCAGATG	160
actB	NM_205518	AAATCAAGATCATTGCCCCACCT	AGGGGTGTGGGTGTTGGTAA	181
gapdh	NM_204305	GCTAAGGCTGTGGGGAAAGT	TCAGCAGCAGCCTTCACTAC	161
rpl4	NM_001007479	TGTTTGCCCCAACCAAGACT	TCCTCAATGCGGTGACCTTT	136
ywhaz	NM_001031343	AGGAGCCCGTAGGTCATCTT	TCCAACAGAGACAGCACATCA	150
gpx4	AF498316	CAGTACAGGGGCTTCGTCTG	CAGCCCCTTCTCAGCGTATC	114
sodmt	NM_204211	CGCTGGCAAAAGGTGATGTT	CTCCTTTAGGCTCCCCTCCT	132

### Statistical methods

Data were subject to mean, t-test and correlation determination using Microsoft Office Excel 2007. Statistical significance was indicated at 0.05% level.

## Results

The average bird weight at hatching (day 1) was 40 g and the final bird weight at day 29 was about 1.3 kg (Table 3). There were no differences in final body weight between the groups for the birds having low and high dietary content of Se and without and with LO (Table 3). The Se concentration was significantly higher in the breast muscle of the animals with the highest Se content in the diet, and the Gpx activity (U/L) in whole blood was also higher in the high Se animals. The breast muscle Se concentration and Gpx activity was not different in animals with and without LO supplementation to the diet (Table 3).

**Table 3:** Final chicken body weight (Final b. w., kg), fat percent of breast muscle (sum of fatty acids, g/100 g tissue wet weight), selenium concentration of breast muscle (mg Se/kg tissue) and Gpx activity (U/L) in whole blood in chickens with low Se in the diet (0.13 mg/kg feed) and high Se (1.1 mg/kg feed) and without or with linseed oil (LO)

	**Selenium**			**Linseed oil**			**Low Se without LO**	**High Se with LO**	
	**Low**	**High**		**Without**	**With**				
	**n = 33**	**n = 32**	***P***	**n = 32**	**n = 33**	***P***	**n = 16**	**n = 16**	***P***
Final b. w.	1.28	1.27	0.842	1.28	1.27	0.600	1.29	1.27	0.604
Fat percent	0.80	0.95	0.109	0.95	0.80	0.091	0.85	0.84	0.913
Se	0.09	0.59	<0.001	0.34	0.33	0.809	0.09	0.59	<0.001
Gpx activity	364	932	<0.001	662	625	0.631	359	897	<0.001

The relative gene expressions are shown in Table 4. The expression of Gpx4 was much higher in the diet groups given high Se content in the feed compared to low Se. Apart from this there were no differences in the gene expression of the antioxidant enzyme Sod, the desaturases Fads1, Fads2, Fads9, HMGCoA reductase, Acox, and Cpt1 between the groups with high and low Se content in the feed. Also, there were no differences in the gene expression of the selected enzymes when the feed was supplemented with LO compared to feed containing no LO. Thus the n-6/n-3 ratio of feed being as low as 1.4 compared to as high as 8.9, and with Se concentrations of 0.13 mg Se/kg feed or 1.1 mg/kg feed did not affect the gene expression of desaturases, HMG-CoA reductase, Acox, Sod, and Cpt1 (Table 4).

**Table 4:** Relative gene expression of fatty acid desaturases Fads1, Fads2, Fads9, HMG-CoA reductase (HMGCoA), (Acox) and (Cpt1), superoxide dismutase (Sod) and glutathione peroxidase4 (Gpx4) in breast muscle of chickens fed low selenium (Se) diet (0.13 mg Se/kg feed) and high Se (1.1 mg Se/kg feed), without or with linseed oil (LO) in the feed

	**Selenium**			**Linseed oil**			**Low Se without LO**	**High Se with LO**	
	**Low**	**High**		**Without**	**With**				
	**n = 33**	**n = 32**	***p***	**n = 32**	**n = 33**	***p***	**n = 16**	**n = 16**	***p***
Fads1	-0.058	0.157	0.054	0.046	0.053	0.954	-0.021	0.201	0.104
Fads2	-0.197	-0.030	0.476	-0.057	-0.170	0.631	-0.001	0.054	0.850
Fads9	-0.126	0.250	0.107	0.022	0.102	0.733	-0.015	0.441	0.216
HMGCoA	-0.046	0.101	0.166	0.052	0.002	0.638	-0.042	0.054	0.453
Acox	-0.011	0.074	0.231	0.010	0.053	0.553	-0.027	0.102	0.197
Cpt1	-0.093	-0.144	0.600	-0.052	-0.185	0.165	-0.024	-0.209	0.257
Sod	0.066	0.142	0.736	0.167	0.041	0.577	0.009	-0.041	0.873
Gpx4	0.011	0.297	<0.001	0.117	0.191	0.358	-0.023	0.337	<0.001

Table 5 shows the fatty acid profile in the chicken breast muscle, given as percent of the total amount of fatty acids in muscle tissue (% FAME). Se supplementation to the feed did not affect the fatty acid profile, except for a small increase in 16:0 and a decrease in 22:6n-3.

**Table 5:** Fatty acid distribution (% of FAME) in chicken breast muscle

	**Selenium**			**Linseed oil**			**Low Se without LO**	**High Se with LO**	
	**Low**	**High**		**Without**	**With**				
**Distribution, % FAME**	**n = 33**	**n = 32**	***p***	**n = 32**	**n = 33**	***p***	**n = 16**	**n = 16**	***p***
C14:0	0.50	0.53	0.262	0.49	0.54	0.052	0.48	0.56	0.028
C14:1 n-5	0.09	0.09	0.167	0.08	0.10	0.006	0.08	0.10	0.004
C15:0	0.14	0.14	0.375	0.13	0.15	<0.001	0.13	0.15	<0.001
C16:0	17.5	17.9	0.038	17.8	17.5	0.068	17.7	17.7	0.863
C16:1 n-7	1.72	1.94	0.095	1.71	1.94	0.091	1.61	2.06	0.016
C17:0	0.29	0.29	0.888	0.27	0.30	<0.001	0.27	0.30	0.002
C18:0	11.0	10.5	0.106	10.6	11.0	0.138	10.7	10.6	0.920
C18:1 n-9	22.4	23.4	0.190	21.9	23.7	0.027	21.5	24.4	0.009
C18:2 n-6	16.8	17.4	0.525	20.8	13.7	<0.001	20.3	13.8	<0.001
C20:0	0.06	0.06	0.394	0.07	0.06	0.116	0.06	0.06	0.650
C18:3 n-6	0.10	0.10	0.999	0.14	0.07	<0.001	0.14	0.08	<0.001
C18:3 n-3	2.87	3.21	0.487	1.27	4.68	<0.001	1.22	4.97	<0.001
C20:1 n-9	0.26	0.26	0.964	0.29	0.23	<0.001	0.29	0.22	0.003
C20:2 n-6	0.75	0.68	0.339	0.93	0.51	<0.001	0.98	0.49	<0.001
C20:3 n-6	0.92	0.82	0.037	0.85	0.89	0.498	0.91	0.84	0.301
C20:3 n-3	0.39	0.36	0.585	0.16	0.57	<0.001	0.17	0.55	<0.001
C20:4 n-6	5.40	4.91	0.359	6.85	3.58	<0.001	7.18	3.43	<0.001
C20:5 n-3	1.42	1.32	0.705	0.37	2.31	<0.001	0.39	2.24	<0.001
C22:5 n-3	2.81	2.50	0.353	1.49	3.75	<0.001	1.61	3.56	<0.001
C22:6 n-3	2.09	1.70	0.044	1.43	2.35	<0.001	1.56	2.09	0.008
20:4n6/20:5n3	9.9	10.0	0.963	18.9	1.6	<0.001	18.8	1.5	<0.001
n-6/n-3	3.6	4.0	0.597	6.4	1.4	<0.001	6.0	1.4	<0.001

Most of the breast muscle fatty acids were highly affected by LO supplementation to the feed (Table 5, middle panel); 14:0, 14:1n-5, 15:0, 17:0, 18:1n-9, 18:3n-3, 20:3n-3, 20:5n-3, 22:5n-3 and 22:6n-3 were significantly increased, and 18:2n-6, 18:3n-6, 20:1n-9, 20:2n-6 and 20:4n-6 were reduced. The combination with low Se without LO were compared to high Se with LO, (Table 5, right panel), and these results were similar to the ‘without LO, and with LO’ groups (middle panel). Thus, the Se supplementation had minor influence, and LO supplementation had the major influence on the fatty acid profile (Table 5).

Product/precursor ratios estimating Delta-9 desaturase activities; shown by the ratio between 16:1n-7/16:0 and 18:1n-9/18:0 and indices of desaturation and elongation in the n-6 and n-3 series of fatty acids in chicken breast muscle from birds fed low or high Se supplemented diets, without or with LO and the combination with low Se/without LO compared to high Se with LO are shown in Table 6. The product/precursor fatty acid indices in meat from the chickens supplemented with low Se were not different compared to the high Se group. LO supplementation, however, had large effects on product/precursor indices. The 16:1n-7/16:0 index increased somewhat in breast muscle with LO compared to without LO supplementation, but the 18:1n-9/18:0 index was not different in breast muscle from birds without and with LO supplementation.

**Table 6:** **Effect of supplementing selenium (Se) and linseed (LO) to the chicken feed on product/precursor ratios between fatty acids; indices for Delta-9 desaturase ( *D-9d *), Delta-6 desaturase ( *D-6d *), Delta-5 desaturase ( *D-5d *), Elongase and combined effects of desaturases and elongases ( *Comb DE *)**

	**Selenium**			**Linseed oil**			**Low Se without LO**	**High Se with LO**	
	**Low**	**High**		**Without**	**With**				
**Product/precursor indices**	**n = 33**	**n = 32**	***p***	**n = 32**	**n = 33**	***p***	**n = 16**	**n = 16**	***p***
*D-9d* 16:1n-7/16:0	0.10	0.11	0.169	0.10	0.11	0.041	0.09	0.12	0.017
*D-9d* 18:1n-9/18:0	2.07	2.28	0.103	2.14	2.20	0.637	2.05	2.33	0.108
*D-6d* 18:3n-6/18:2n-6	0.006	0.006	0.747	0.006	0.005	<0.001	0.007	0.005	0.008
*D-5d* 20:4n-6/20:3n-6	6.00	6.09	0.883	8.15	4.06	<0.001	8.08	4.09	<0.001
*Elongase* 20:3n-6/18:3n-6	10.52	9.38	0.320	6.89	12.86	<0.001	7.08	11.89	<0.001
*Comb DE* 20:3n-6/18:2n-6	0.06	0.05	0.086	0.04	0.07	<0.001	0.05	0.06	<0.001
*Comb DE* 20:4n-6/18:2n-6	0.32	0.28	0.087	0.34	0.26	<0.001	0.36	0.25	<0.001
*Elongase* 22:5n-3/20:5n-3	2.88	2.78	0.768	4.10	1.64	<0.001	4.16	1.60	<0.001
*Comb DE* 20:5n-3/18:3n-3	0.47	0.40	0.212	0.33	0.54	<0.001	0.36	0.50	0.042
*Comb DE* 22:5n-3/18:3n-3	1.23	1.01	0.181	1.36	0.90	0.003	1.49	0.81	0.005
*Comb DE* 22:6n-3/18:3n-3	1.03	0.83	0.256	1.32	0.57	<0.001	1.43	0.48	<0.001
*Comb DE* 22:6n-3/20:5n-3	2.55	2.27	0.499	3.90	1.03	<0.001	4.08	0.93	<0.001
*Comb DE* 22:6n-3/22:5n-3	0.81	0.75	0.275	0.95	0.62	<0.001	0.97	0.59	<0.001
20:5 + 22:5 + 22:6/18:3n-3	2.73	2.24	0.175	3.01	2.01	0.004	3.27	1.79	0.003

The index for the n-6 PUFAs delta-6 desaturase 18:3n-6/18:2n-6, delta-5 desaturase 20:4n-6/20:3n-6 and the index 20:4n-6/18:2n-6 was lower in the breast muscle from the birds given supplement of LO in the feed compared to no LO.

The index for n-3 PUFAs 22:5n-3/18:3n-3, 22:6n-3/18:3n-3, 22:6n-3/20:5n-3, 22:6n-3/22:5n-3 and (20:5n-3 + 22:5n-3 + 22:6n-3)/18:3n-3 were all highly reduced compared to breast muscle from chickens without LO (Table 6).

The indices that estimated elongase activities: the n-6 C18 product-precursor ratio 20:3n-6/18:3n-6 and the n-3 ratio 22:5n-3/20:5n-3 were different when LO was supplemented to the feed compared to without LO; being higher for the C18 n-6 PUFA and lower for the C20 n-3 PUFA (Table 6).

The indices in muscle from the animals fed with low Se without LO compared to high Se with LO, (Table 6, right panel), were equal to the results for the animals fed without and with LO (Table 6, middle panel).

### Associations between expressions of genes involved in fat and antioxidant metabolism and content of fat, fatty acids and fatty acid indices in the diet group ‘low Se-without LO’

Table 7 shows coefficients of correlation between the expressions of individual genes involved in fat metabolism in breast muscle from 16 chickens fed a diet low in Se and without LO and fatty acids. There were no associations between Fads1, 2 and 9 and the respective indices for Delta-5 desaturase, Delta-6 desaturase and Delta-9 desaturase. There were some positive associations between the various gene expressions; Fads1 correlated positively to the expression of HMG-CoA reductase, Acox, Sod and Gpx4. Fads2 correlated positively to the expression of Fads9. Gene expression of HMG-CoA reductase correlated positively to Acox, and Gpx4. Acox correlated positively to Cpt1 and Gpx4. The fat percent in the muscle did not show any correlations to the gene expressions (Table 7). There were not many significant correlations between the expressions of Fads1, Fads2, Fads9, HMGCoA reductase, Acox, Cpt1, Sod and Gpx4 and percent of fatty acids in chicken breast muscle, (and also when presented as mg fatty acids/g tissue wet weight, data not shown).

**Table 7:** Association (correlation coefficients) between expressions of the genes Fads1, Fads2, Fads9, HMGCoA reductase, Acox, Cpt1, Sod, Gpx4 and fat percent of breast muscle and final body weight (Final b.w.) from chickens (n = 16) supplemented with low selenium (Se) and without linseed (LO) in the feed, fatty acid indices and fatty acids

**n = 16**	**Fads1**	**Fads2**	**Fads9**	**HMGCoA**	**Acox**	**Cpt1**	**Sod**	**Gpx4**	**Fat %**
Fads1	1								
Fads2	-0.324	1							
Fads9	-0.314	0.833*	1						
HMGCoA red	0.492*	-0.299	-0.115	1					
Acox	0.592*	0.207	0.272	0.516*	1				
Cpt1	-0.061	0.320	0.361	0.153	0.506*	1			
Sod	0.719*	-0.171	-0.340	0.189	0.427	-0.116	1		
Gpx4	0.550*	-0.399	-0.199	0.560*	0.677*	0.445	0.359	1	
Fat%	0.225	-0.161	0.114	0.367	0.064	-0.213	0.072	0.147	1
*D-9d* 16:1n-7/16:0	0.140	-0.056	0.143	0.245	-0.013	0.005	-0.025	0.214	0.813*
*D-9d* 18:1n-9/18:0	0.194	-0.149	0.110	0.256	-0.051	-0.169	-0.054	0.121	0.908*
*D-6d* 18:3n-6/18:2n-6	0.277	-0.169	-0.080	0.458	0.117	0.143	0.289	0.328	0.714*
*D-5d* 20:4n-6/20:3n-6	-0.165	-0.223	-0.348	-0.378	-0.260	-0.034	0.116	-0.059	-0.283
*Elongase* 20:3n-6/18:3n-6	-0.210	0.289	0.099	-0.258	0.066	-0.096	-0.104	-0.260	-0.740*
*Comb DE* 20:3n-6/18:2n-6	-0.144	0.307	0.031	-0.112	0.104	0.023	0.003	-0.182	-0.743*
*Comb DE* 20:4n-6/18:2n-6	-0.238	-0.019	-0.346	-0.388	-0.163	-0.050	0.148	-0.169	-0.807*
*Elongase* 22:5n-3/20:5n-3	-0.012	0.062	-0.129	-0.248	-0.113	-0.025	0.175	-0.176	-0.224
*Comb DE* 20:5n-3/18:3n-3	-0.339	0.132	-0.080	-0.344	-0.123	-0.142	-0.008	-0.277	-0.767*
*Comb DE* 22:5n-3/18:3n-3	-0.236	0.050	-0.237	-0.405	-0.186	-0.223	0.151	-0.307	-0.731*
*Comb DE* 22:6n-3/18:3n-3	-0.186	0.068	-0.196	-0.379	-0.092	-0.164	0.206	-0.248	-0.694*
*Comb DE* 22:6n-3/20:5n-3	0.066	0.090	0.018	-0.127	0.102	0.144	0.218	0.007	-0.087
*Comb DE* 22:6n-3/22:5n-3	0.165	0.107	0.223	0.055	0.357	0.311	0.217	0.248	0.127
20:5 + 22:5 + 22:6/18:3n-3	-0.230	0.067	-0.208	-0.396	-0.142	-0.193	0.163	-0.285	-0.734*
Final b.w.	0.373	-0.530*	-0.448	0.074	-0.103	-0.201	0.092	0.317	0.023
Se mg/kg	-0.413	-0.120	-0.156	-0.199	-0.228	0.269	-0.403	0.052	-0.107
Gpx U/l	0.020	0.120	0.299	-0.190	-0.062	-0.264	-0.131	-0.352	0.264
% FAME									
C14:0	0.292	-0.375	-0.065	0.329	-0.112	-0.281	0.041	0.083	0.851*
C14:1 n-5	0.249	-0.192	0.068	0.329	-0.001	-0.116	0.008	0.243	0.910*
C15:0	0.364	-0.536*	-0.298	0.421	-0.059	-0.211	0.231	0.254	0.746*
C16:0	0.107	-0.342	-0.215	0.329	-0.244	-0.359	-0.066	-0.201	0.189
C16:1 n-7	0.161	-0.099	0.117	0.302	-0.038	-0.039	-0.033	0.191	0.839*
C17:0	0.438	-0.462	-0.379	0.500*	0.323	0.234	0.348	0.538*	0.487*
C18:0	-0.197	0.107	-0.175	-0.307	0.037	0.154	0.112	-0.100	-0.856*
C18:1 n-9	0.188	-0.147	0.100	0.236	-0.042	-0.059	-0.075	0.161	0.858*
C18:2 n-6	0.234	0.103	0.443	0.368	0.325	0.127	-0.183	0.244	0.761*
C20:0	-0.030	-0.551*	-0.472*	0.166	-0.264	-0.258	-0.044	-0.083	0.156
C18:3 n-6	0.304	-0.125	0.060	0.494*	0.180	0.113	0.193	0.333	0.861*
C18:3 n-3	0.259	-0.204	0.102	0.328	-0.011	-0.098	-0.001	0.197	0.928*
C20:1 n-9	0.036	0.109	-0.092	0.096	0.175	0.298	0.041	0.108	-0.672*
C20:2 n-6	0.265	0.292	0.043	0.210	0.490*	0.204	0.287	0.089	-0.672*
C20:3 n-6	-0.086	0.405	0.202	-0.006	0.239	0.111	-0.069	-0.114	-0.611*
C20:3 n-3	0.268	0.355	0.159	0.150	0.479*	0.094	0.279	-0.021	-0.608*
C20:4 n-6	-0.245	0.078	-0.233	-0.361	-0.040	0.083	0.083	-0.105	-0.861*
C20:5 n-3	-0.435	0.117	0.020	-0.292	-0.185	-0.084	-0.159	-0.274	-0.687*
C22:5 n-3	-0.306	0.060	-0.193	-0.415	-0.248	-0.162	0.096	-0.342	-0.762*
C22:6 n-3	-0.194	0.094	-0.084	-0.345	-0.059	-0.013	0.194	-0.202	-0.635*

*P < 0.05 when correlation coefficient >0.468 or < -0.468.

The fat percent in the breast muscles varied from 0.6 to 1.5% in the 16 animals, and the fat percent was strongly positively correlated to the indices for Delta-9 desaturase (16:1n-7/16:0 and 18:1n-9/18:0) and the Delta-6 desaturase (18:3n-6/18:2n-6) (Table 7). Quite the opposite, there were strong negative associations between fat percent in the muscle and the estimate for elongase 20:3n-6/18:3n-6 and the combined effects of elongases and desaturases for fatty acids produced from both LA and ALA (Table 7). The fat percent associated positively to all fatty acids from C14 up to C20, and negatively to the percent of the C20 and C22 n-6 and n-3 PUFAs. Correlations were also calculated for the other three diet groups of animals (data not shown), giving about the same results as shown in Table 7.

## Discussion

The aim of the present study was to compare the indices (product/precursor ratios) estimating desaturases and elongases participating in the formation of MUFAs, and n-6 and n-3 LCPUFAs with the expression of genes involved in lipid metabolism, and with fat percent in breast muscle of chickens fed diets with or without linseed oil and high and low selenium. Desaturases and elongases are important regulators in metabolism of fatty acids and they are expressed in many tissues, e.g. in skeletal muscle [26,27]. The expression of genes involved in fat metabolism are regulated by nutrients such as minerals, vitamins and fatty acids, hormones and other environmental factors [26,27]. To our knowledge, there is little, if any, information of comparison of the expression of genes in fat metabolism with indices of elongases and desaturases and with individual fatty acid profiles and fatty concentrations in muscle from chickens fed diets enriched in ALA and Se.

In the present study, the diets with high and low Se supplementation showed that high Se supplementation did not cause large differences in fatty acid profiles in chicken breast muscle.

Linseed oil, on the contrary, resulted in large differences in fatty acid profiles in chicken muscles; significantly increased levels of ALA, EPA, DPA and DHA, and reduced content of LA and AA, compared to muscle from chickens without LO in the feed. The product/precursor indices for fatty acids were highly significantly different for desaturation and elongation of ALA to EPA, DPA and DHA, and LA to AA between the diet groups without or with LO. The differences in indices of product/precursor fatty acid were not a result of changes in gene expression since the gene expression of enzymes participating in desaturation and in general lipid metabolism was similar in the diet groups without and with LO.

The decreased Delta-6 and Delta-5 desaturase activity in the LO supplemented diet, estimated by product/precursor indices are in line with studies on fatty acid desaturase activity in erythrocytes that were decreased due to low n-6/n-3 ratios [28,29]. In the present study, the animals that were given a diet with LO had an n-6/n-3 ratio on 1.4 in breast meat and the ratio was 6.4 in breast meat from the ‘without LO’ group. Since the product/precursor estimate for the two elongase reactions 20:3n-6/18:3n-6 and 22:5n-3/20:5n-3 showed opposite responses to LO supplementation, it could be suggested that two different elongases (Elovl2 and Elovl5) might be the main enzymes in these two conversions. But this suggestion has to be verified by direct enzyme measurements.

The sum of EPA + DPA + DHA in the chicken breast muscle was 0.3 mg/g in the group without LO, and 0.7 mg/g in the LO supplemented group. The AA content was 0.6 mg/g and 0.3 mg/g in the two groups, respectively, thus the sum of n-3 + n-6 LCPUFAs were close to identical in the groups without and with LO supplementation. The sum of LA + ALA was 2.3 mg/g and 1.6 mg/g muscle tissue in group without and with LO supplementation, respectively. Since the animals did not have any n-3 and n-6 LCPUFAs in the diet during their growth from 40 g at hatching till 1.3 kg at slaughtering, it must be anticipated that AA, EPA, DPA and DHA was produced by endogenous conversion of ALA to n-3 LCPUFAs and LA to AA.

The n-3 and n-6 fatty acids compete for the same desaturase and elongase enzymes in the fatty acid pathway from LA to AA and from ALA to EPA + DPA + DHA. Since the sum of n-3 + n-6 PUFAs in breast muscle was lower in muscle of the chickens fed with LO compared to without LO, the total need for elongases and desaturases could be anticipated to be less in the group ‘With LO’ compared to ‘Without LO’. However, the gene expression of Fads1 and 2 were the same in breast meat from chickens fed diets with or without LO (Table 4). These findings are in line with the study by Tu et al. [18] in rats that were fed diets containing between 0.2 and 2.9 energy percent ALA, showing no differences in expression levels of desaturases and elongases between the diet groups (in the present study the energy percent from ALA was 0.7% in the feed without LO, and 2.5% in the feed with LO).

The expression of Fads9 in chicken breast muscle was not affected by supplementation of Se or LO to the feed. Delta-9 desaturase indices estimated by the ratios between 16:1n-7/16:0 and 18:1n-9/18:0 in breast muscle showed that the indices were not changed by Se supplementation, but the 16:1n-7/16:0 index was slightly higher in the ‘With LO’ group, compared to ‘Without LO’. Delta-9 desaturase is known to be inhibited by PUFA [4]. The sum of total PUFAs in the breast muscle in the ‘With LO’ group was 2.8 mg/g, and it was 3.4 mg/g in the ‘Without LO’ muscle. It might be speculated that this difference in PUFA caused the higher 16:1n-7/16:0 Delta-9 desaturase index in the group supplemented with LO.

The Se concentration was either 0.13 mg/kg or 1.1 mg/kg feed, which is at the lower and higher range of Se intake for chickens. The birds having 0.13 mg Se/kg feed or 1.1 mg Se/kg had a normal growth, and had not higher mortality than normal, indicating that the intake was acceptable. The recommended lowest intake is 0.15 mg/kg feed for poultry, and highest recommended intake is 0.5 mg/kg [30].

Comparison of desaturase indices in muscle from the animals fed with low Se without LO to high Se with LO, (Table 6, right panel), with animals fed without and with LO (Table 6 middle panel), shows that LO had major effects on the indices, and Se supplementation had only minor effect on the indices.

It has been shown that a high intake of ALA resulted in a relatively lower conversion of ALA to n-3 LCPUFAs compared to a diet with less ALA [12,31], and this is confirmed in the present study: The indices for desaturation and elongation of ALA to the sum EPA + DPA + DHA, and ALA to DPA and DHA in the LO fed animals showed a decrease compared to the group fed without LO (Table 6), indicating that the LO supplementation slowed down the formation to n-3 LCPUFAs compared to a diet containing less ALA. The fatty acid pathway from LA to AA; the index AA/LA was reduced in the breast muscle from chickens supplemented with LO (Table 6), showing that the formation of AA was also reduced by the supplementation of ALA. LO supplementation resulted in more than a doubling of n-3 LCPUFAs in breast muscle. Since the expression of the measured genes in lipid and antioxidant metabolism was not affected by the LO supplementation in the present study, this indicates that the profile of n-3 LCPUFAs in chicken muscle may be controlled through substrate competition, feed-back control and esterification rather than by alterations in gene expression of enzymes in lipid metabolism such as Fads1, Fads2, Fads9, HMGCoA reductase, Acox and Cpt1. This is consistent with findings in rats [18].

Breast muscle Se content, Gpx activity in whole blood and Gpx4 expression were all increased by high Se intake. The increased expression of the Gpx4 gene with high Se intake indicates an up-regulation when Se intake is increased from 0.13 to 1.1 mg/kg feed. An increase in Gpx4 expression following Se supplementation in chicken muscle has also been shown by others [32].

### Associations between fatty acid indices, fat percent and expression of genes in lipid metabolism in breast muscle from the birds fed low Se and without LO

Noteworthy, as shown in Table 7, there is a strong positive correlation between muscle fat content and synthesis of MUFA as estimated by 16:1n-7/16:0 and 18:1n-9/18:0 indices, and a strong negative correlation between muscle fat and synthesis of AA, EPA, DPA, DHA and DGLA. The fat percent in muscle has been shown to be a determinant for metabolic disease, inflammation and non-chronic diseases [22]. Indeed, the present results confirm that there are strong correlations between fat percent and indices for desaturases and elongases, apparent supporting the role of muscle fat percent as a marker in relation to chronic diseases in man. However, studies in humans are required to substantiate this hypothesis.

The lack of correlations between muscle fat percent and expression of genes in lipid metabolism is surprising. There were no correlations between gene expression of Fads1 and the index estimate of Delta-5 desaturase 18:3n-6/18:2n-6, and no correlation between expression of Fads2 and the estimate of Delta-6 desaturase 20:4n-6/20:3n-6, as shown in Table 7 in the ‘low Se-without LO’ group, but also the same was shown when calculated on the other animals in the study. A positive association between Fads9 and the estimates of Delta-9 desaturase indices 16:1n-7/16:0 and 18:1n-9/18:0 were expected, but as shown in Table 7, the association did not reach level of significance. The lack of association between the gene expression for the fatty acid desaturases and the corresponding product/precursors ratios confirms the role of fatty acid concentrations, and not gene expressions, as the main regulators of the enzyme activities.

As shown in Table 7, the fat percent of the muscle was positively associated with percent of most fatty acids with 14 to 18 C-atoms (not 18:0), and negatively associated with 18:0 and all the C20 and C22 unsaturated fatty acids. Thus, the amount of fat in the muscle is fundamental for the fatty acid profile of meat; much fat in muscle results in lower percentage of LCPUFAs. This is also shown and discussed by others [33,34].

The positive correlation between the expression of the genes for Fads1, HMGCoA reductase, Acox, Cpt1 and Sod indicates that there may be a link between these genes, possibly via a central regulation of the gene expression. It is known that many genes related to lipid metabolism are controlled by the same transcription factors such as peroxisome proliferator-activated receptor (PPAR) and sterol response element binding protein (SREBP) [20,35]. Many reactive oxygen species are continuously produced as byproducts of aerobic metabolism. Sod is connected to β-oxidation of fatty acids by catalyzing the conversion of reactive oxygen species produced during β-oxidation to hydrogen peroxide [36].

The expression of HMGCoA reductase was positively correlated to the Acox and Gpx4 expression. HMGCoA reductase is involved in cholesterol, coenzyme Q10 and dolichole synthesis. Coenzyme Q10 has a double function, it is an essential part of the electron transport system in the mitochondrial respiratory chain but it is also an important endogenous antioxidant, especially in its reduced form (ubiquinol). It has a rapid turnover in mammals, presumably because of rapid peroxidative degradation of its side chain with numerous double bonds. With higher dietary intake of LO rich in ALA, it could be suggested that the synthesis of the antioxidant ubiquinol was up-regulated, but no effect was observed on expression of HMGCoA reductase in the present study (Table 4).

The positive association between Acox and the expression of Cpt1 and Gpx4 may indicate a link to mitochondrial β-oxidation and antioxidant status, but the lack of effect on gene expression of Acox by Se and LO, and the lack of association between Acox and other fatty acids and fatty acid ratios indicates that other factors than the Se content and the LO intake affected Acox.

## Conclusion

Linseed oil in the diet had a large impact on fatty acid concentrations in chicken breast muscle; resulting in more ALA, EPA, DPA and DHA and less LA and AA.

The expression of genes in lipid metabolism did not seem to be affected by supplementation with Se and LO to the feed, indicating that the concentration of LCPUFAs may be regulated independently of changes in the expression of the relevant enzymes, and possibly more by the substrate competition.

The positive correlation between expression of selected genes involved in lipid and antioxidant biosynthesis may indicate a co-ordinate regulation of these genes.

The strong positive association between fat percent in breast muscle and the desaturase indices for production of the MUFAs 16:1n-7 and 18:1n-9, and negative associations between fat percent and indices for production of the LCPUFAs AA, DGLA, EPA, DPA and DHA is interesting since these fatty acids have been linked to health conditions. Whether the associations between muscle fat and these fatty acids are a causal relationship should be further studied.

## Competing interests

The authors declare that they have no competing interests.

## Authors’ contributions

The present study is conceived and conducted by AH and NFN. TKØ analyzed the gene expressions and all authors analyzed and interpreted the data, and drafted the article. All authors approved the final version to be published.
